# Robotic-Assisted Splenectomy by a Modified Lateral Approach: Technique and Outcomes

**DOI:** 10.7759/cureus.43820

**Published:** 2023-08-20

**Authors:** Pottakkat Biju, Ram Prakash Gurram, Raja Kalayarasan, Pothugunta S Krishna

**Affiliations:** 1 Surgical Gastroenterology, Jawaharlal Institute of Postgraduate Medical Education & Research, Puducherry, IND

**Keywords:** pancreatic fistula, modified lateral approach, hematological diseases, total splenectomy, robotic-assisted surgery

## Abstract

Introduction

The utilization of robot-assisted technique for splenectomy has recently gained popularity especially in patients undergoing splenectomy for hematological indications owing to its magnification of and easy manipulation of internal abdominal organs. Moreover, robotic splenectomy emerged as an essential teaching module before approaching more complex robotic procedures.

Methods

A total of 43 elective splenectomies were performed for hematological indications in Department of Surgical Gastroenterology, Jawaharlal Institute of Postgraduate Medical Education and Research (JIPMER) between January 2018 to July 2023 of which 14 patients underwent robotic splenectomy. All patients underwent lateral approach of robotic splenectomy with a modification of avoiding the lesser sac dissection. Prospectively maintained data were retrospectively analyzed and results were recorded in terms of intra-operative time taken, blood loss, need for blood and blood product transfusion and postoperative morbidity and mortality.

Results

The indications for patients who underwent robotic splenectomy include idiopathic thrombocytopenic purpura in eight patients, autoimmune hemolytic anemia in three patients, Evans syndrome in one patient and hereditary spherocytosis in two patients. The median splenic diameter was 14.8cm and the median platelet count before the operation was 10,800 cells/cubic millimeter (7000-3,20,000). The mean operative time was 92 minutes and blood loss was 40ml. The median duration of hospital stay was 2.4 days. All 14 patients had therapeutic success and there was no procedure-related mortality or morbidity.

Conclusion

Robotic splenectomy using the modified lateral approach can safely be performed with comparable operative time, blood loss and overall morbidity. However further studies are mandatory to confirm the advantage of this modified technique of lateral approach of robotic splenectomy.

## Introduction

A minimally invasive approach has become the standard approach for splenectomy, especially in haematological conditions where splenectomy is indicated [1]. Robotic surgery since its introduction is considered as an advanced version of laparoscopy owing to its inherent advantages and gained widespread acceptance in various surgical fields. Similarly, robotic surgery platform is being increasingly utilized to perform splenectomy [2,3]. The reported advantages of robotics in performing splenectomy are owing to its magnification and maneuverability [3]. Traditionally, splenectomy is performed by two approaches: lateral approach and medial approach. The superiority of one technique over the other is a subject of debate since both approaches have well-known limitations [4-6]. This distinction is less clearly understood and is not accurately described in the scientific literature pertaining to robotic-assisted splenectomy. With most of the complications related to minimally invasive splenectomy being related to technique, it is important to practice and apply the accurately described technique to avoid any mishaps [7]. Traditionally, much importance is given to opening the lesser sac widely and dissecting the splenic vessels thoroughly. In this study, we aim to describe a total lateral approach using robotic-assisted splenectomy using lateral ports with a technical modification where entering the lesser sac is completely avoided.

## Materials and methods

The study was conducted after obtaining approval from the institute's research board committee. Prospectively maintained data were retrospectively analyzed for the study period of January 2018 to July 2023. Minimally invasive splenectomy was a natural choice except in cases of trauma. The decision of whether to subject the patient to robotic approach was the surgeon's and patient’s choice. Preoperative platelet transfusion was advocated only selectively based on various clinical and laboratory parameters [8].

Technical report

We describe our technique under the following headings: Anaesthesia and patient positioning; Port placement; Division of spleno-colic ligament; Exposure to the splenic hilum; Division of splenic hilum; Delivering the specimen.

Anaesthesia and Patient Positioning in Right Lateral Decubitus With Left Arm Abducted

**Figure 1:**
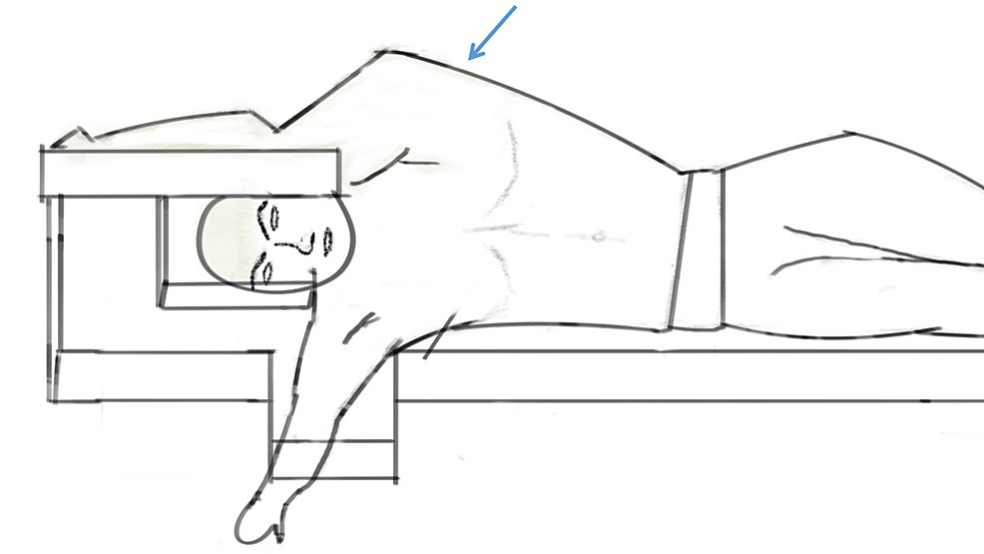
Patient positioning: Right lateral decubitus position with left arm abducted away from the operating field (blue arrow).

All surgeries were performed under general anaesthesia. The patient is positioned in right lateral decubitus with the left arm abducted away from the surgical field. A flank cushion or a bean bag is placed under the right flank to widen the space between the subcostal margin and the iliac crest. Adequate widening of the space is important to create ample working space inside the abdominal cavity in the robotic approach considering the length of the robotic arms (Figure 1).

Port Placement

**Figure 2:**
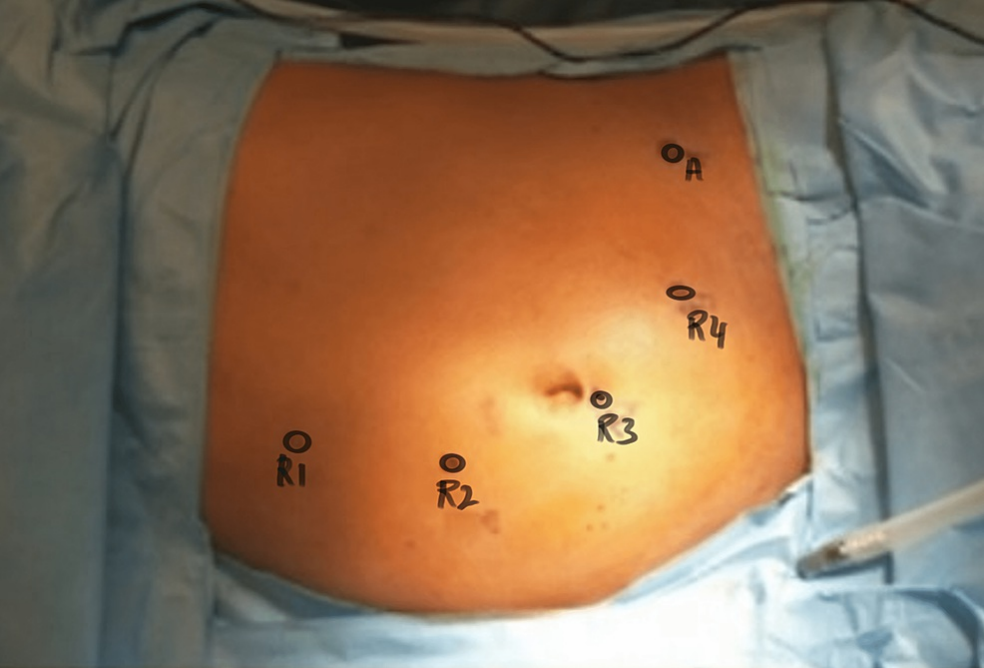
Robotic arm port positioning R1 - robotic arm 1 R2- robotic arm 2 R3 - robotic arm 3 R4 - robotic arm 4 A - Assistant port

The da Vinci Xi system (Intuitive, Sunnyvale, CA, USA) was used for operation. The first port, which is R3, is inserted at the infra-umbilical site. The pneumoperitoneum is created and the abdomen is slowly inflated till a set pressure of 10-12mm Hg is reached with a flow rate of 5-6 Liters per minute. The remaining ports are placed under vision in a straight diagonal line parallel to the splenic axis maintaining approximately 15cm from the spleen. One port is placed above the umbilicus level and two ports are below it much laterally near the posterior axillary line. One 12mm external assistant port is placed in between the third and fourth robotic ports or lateral to the fourth robotic port. Once the ports are positioned in the desired location, the robot is docked from the left side of the patient. Targeting is done over the splenic hilum. Camera in R3, Cadiere forceps in R1 and fenestrated bipolar forceps in R2 of the robotic arms respectively are used. In R4 monopolar scissors (MS) or robotic EndoWrist cautery hook are used (Figure 2).

Division of Splenocolic Ligament

**Figure 3:**
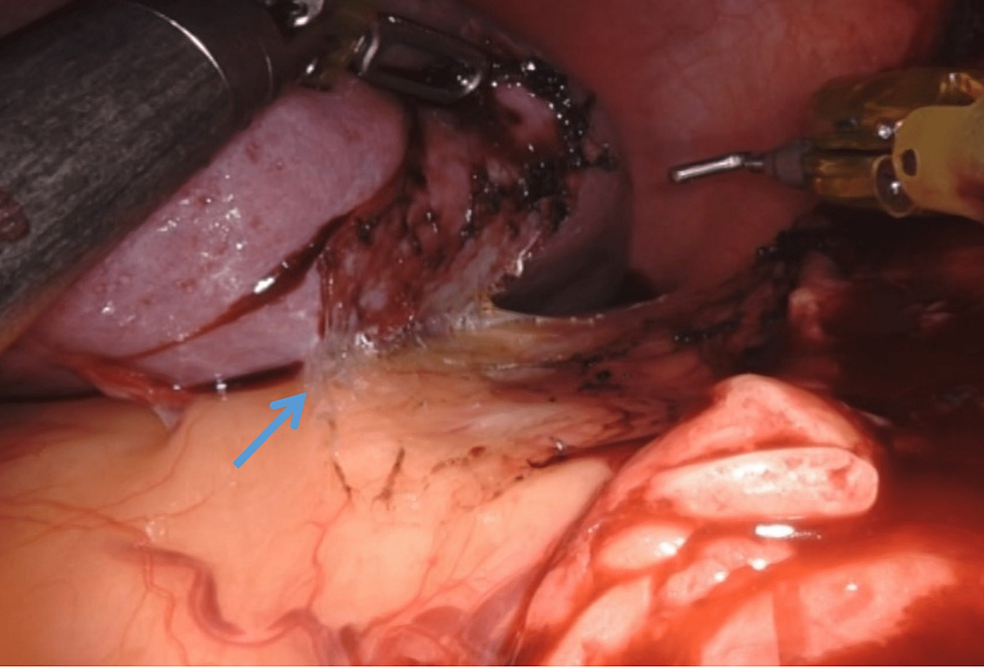
Robotic view of division of splenocolic ligament: the initial step of dissection (blue arrow).

The dissection in the lateral approach starts with the division of the splenocolic ligament. The degree of angulation of splenic flexure determines the difficulty of this step. However, it is simplified owing to the degree of freedom offered by the robotic system (Figure 3). The position of the patient themself suspends the spleen from its attachments facilitating dissection. Gravity in the right lateral decubitus position offers the traction needed from the colonic side while gentle anterior traction of the spleen with fenestrated bipolar provides the necessary counter-traction. Once the inferior pole of the spleen is seen it is mobilized along with the posterior attachment of the spleen till the division of the spleno-phrenic ligament. This complete lateral mobilization is possible with robotic instruments, unlike laparoscopic instruments. Now spleen has only medial attachments.

Exposure to the Splenic Hilum

**Figure 4:**
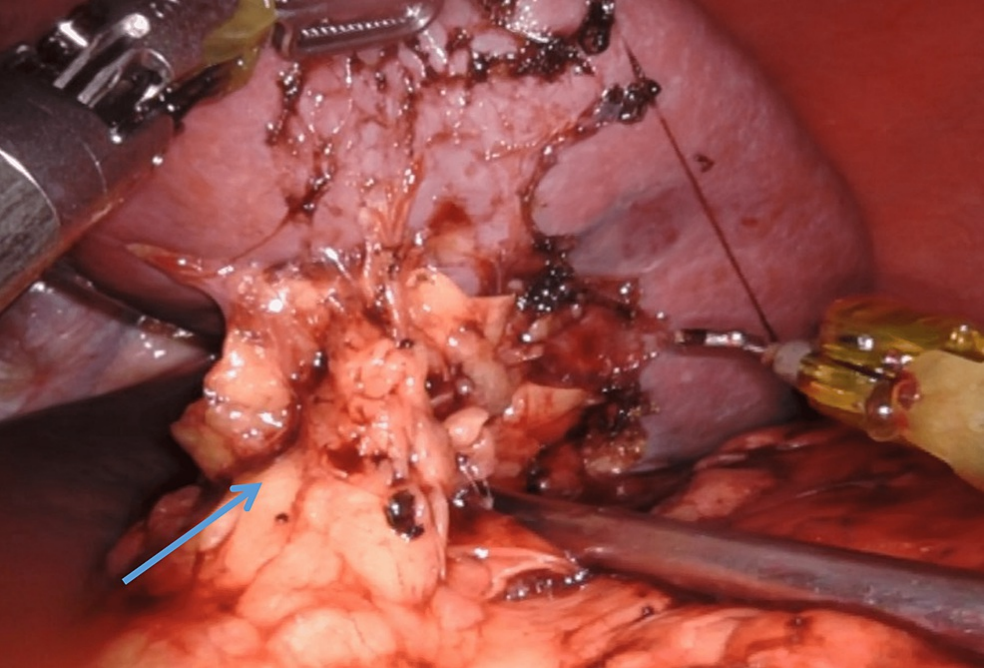
Dissection near the splenic hilum initiating from the superior pole splenic capsule avoiding gastrocolic ligament dissection (blue arrow).

A window is created just near the splenic capsule at the superior pole of the spleen above the hilum and the short gastric vessels are divided except the last vessel at the tip of the fundus which will be divided at the end of the dissection (Figure 4). Unlike the conventional approach, the gastrocolic omentum is not divided to enter the lesser sac to approach splenic vessels.

Division of Splenic Hilum

**Figure 5:**
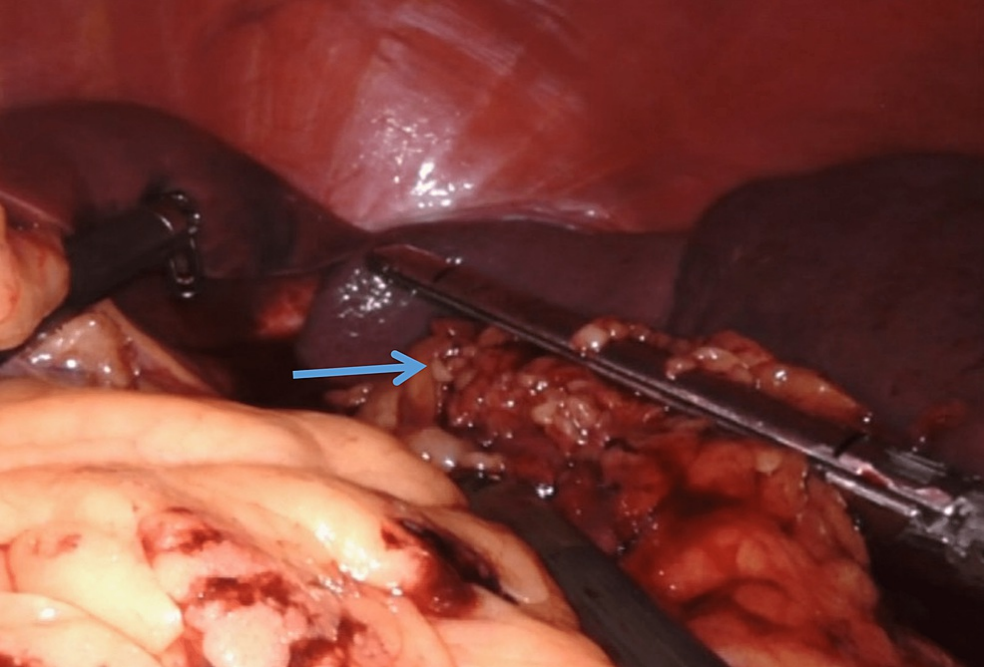
Robotic pictorial depiction showing endostapler division of splenic hilum (blue arrow).

The robotic instrument is passed below the terminal part of the tail of the pancreas slowly and delivered to the upper border of the splenic hilum. Vessels are not individually separated or skeletonized. We prefer the application of a vascular endostapling device after visualising the tail of the pancreas, through the assistant port as it addresses both the concentrated type and distributed type of splenic arterial anatomy effectively (Figure 5) [9]. After the division of the splenic hilum, the last short gastric vessels are divided, and the spleen is left free inside the abdomen for retrieval.

Delivering Specimen

The assistant port or the lateral most robotic arm port is converted to a 15mm port and an endobag is introduced and the spleen is placed inside the bag. In some patients, once the spleen is placed inside the endobag through the 12mm assistant port, the port incision is extended to 3cm. The spleen is morcellated and removed to prevent any spillover into the abdominal cavity. We don't prefer placing an abdominal drain.

## Results

From January 2018 to July 2023, 43 patients underwent splenectomy for hematological indications. Among them, 14 underwent robotic splenectomy using the modified technique, 24 underwent laparoscopic splenectomy and five underwent open splenectomy.

The mean age of the 14 operated patients was 29.6 years which included 10 males and four females with a mean BMI of 23.2kg/m (19-28). All patients had haematological disorders, among which eight patients were diagnosed with idiopathic thrombocytopenic purpura, three patients with autoimmune hemolytic anaemia, one with Evans syndrome and two patients with hereditary spherocytosis. The median splenic diameter was 14.8 (13-16) cm and the median platelet count before the operation was 10,800 cells/cubic millimetre (7000-3,20,000). The preoperative parameters are reported in the Table 1.

**Table 1:** Description of preoperative parameters.

Preoperative parameters	Number of patients (N=14)
Males/Females	10 patients/4 patients
Mean age of patients	29.6 years
Mean body mass index	23.2 Kg/square metre
Mean splenic diameter	14.8 cm
Mean platelet count before splenectomy	10,800 cells/cubic millimeter
Indication for splenectomy - Idiopathic thrombocytopenic purpura	8 patients
Autoimmune hemolytic anemia	
	3 patients
Hereditary spherocytosis	2 patients
Evans syndrome	
	1 patient

There was no procedure-related mortality. There was no conversion to either laparoscopic or open procedure. The mean time taken for docking was six minutes (four to 10 minutes). The mean operative time was 92 minutes (65 to 160 minutes) and blood loss was 40ml (20 to 150ml). Splenic hilum was divided by an endostapling device in all cases. None of the patients required intraoperative blood or blood products transfusion. Postoperatively one patient required platelet transfusion. There was no re-exploration. The median duration of hospital stay was 2.4 days (two to three days). All 14 patients had therapeutic success defined by improvement of platelet count to greater than 50000/mm3 within four to six weeks post surgery. None of our patients developed postoperative pancreatic fistula. The intraoperative parameters and postoperative outcomes are reported in Table 2.

**Table 2:** Depiction of intraoperative parameters and postoperative outcomes.

Intraoperative parameters and postoperative outcomes	
Mean time for docking	6 minutes
Mean operative time	92 minutes
Mean blood loss	40 ml
Need for intraoperative blood transfusion	None
Need for postoperative platelet transfusion	One patient
Mean duration of hospital stay	2.4 days
Therapeutic success (defined by platelet count more than 50000 cells/cubic millimeter)	14 patients (100%)
Pancreatic fistula	None
Mortality	None

## Discussion

The results of our study correlate closely with those of previous studies which have employed robotic platforms for splenectomy although via different approaches [10,11]. The feasibility of robotic-assisted splenectomy has been established in previous studies with robot-assisted surgeries having advantages over the laparoscopic approach, particularly in challenging clinical situations [11]. Pier et al. reported the initial case report of robotic partial and total splenectomy in 2011 and included 24 patients. The authors reported a mean operative time of 199 ± 65 minutes, mean blood loss of 75ml and mean hospital stay of 5.5 days. The mean operating time and hospital stay were less in our series. However, the study also reported conversion to open procedure in two patients due to splenic artery haemorrhage and an overall morbidity of 8.3%. Patients in our study had no intra-operative or post-operative complications. The decreased extent of manipulation of abdominal organs in robotic surgery combined with discrete, stable vision and absolute precision is expected to further bring down the complication rate associated with any procedure and splenectomy is no exception for the same [12]. Data regarding robot-assisted splenectomy is emerging as shown in a recent meta-analysis [13] with many institutions initiating the acquisition of robots into their therapeutic armamentarium. It is expected to give more insights into the application of robots to the field of splenectomy to pinpoint specific indications of this relatively costly procedure where it can be of more help. With robot-assisted splenectomy propagated as a bridge to train surgeons [13] to perform more complex robotic surgeries, technical reports with accurate descriptions of procedures may play a crucial role in imparting confidence to the surgeon by serving as a guided navigational system.

All our patients had haematological disorders and the new technique offers advantages in this subgroup by avoiding division of gastro-colic ligament, avoiding dissection through lesser sac and avoiding individual skeletonization of splenic vessels. The lateral approach was preferred over the medial approach owing to various factors like better visualization of splenic hilum, ease of dissection in pancreatic capsule away from splenic hilum if needed, the aid of gravity offered in the right lateral position avoiding the need for any additional retraction, access to the accessory spleens if needed [14]. The position of the patient and the lateral port placements are important to effectively execute this technique. Unlike the conventional technique where the focus is done looking at the splenic hilum from the right and anterior, in this modified lateral technique, the focus is done looking at the splenic hilum from the inferior and posterior ignoring the gastrocolic ligament. The whole concept of this modified technique is aimed to divide all attachments including vessels close to the spleen avoiding all avoidable anatomical dissections near the stomach, pancreas and splenic vessels with robotic arms with an exceptional degree of freedom of movement to help the surgeon approach any area around the spleen under vision. This has the potential to give dividends in patients with haematological diseases with low hemoglobin and low platelet counts. These intraoperative advantages translate into improved postoperative outcomes as well [15]. Our technique resulted in less operative time, and less hospital stay with no complications. Two out of four ports are placed near posterior axillary line and this gives an additional cosmetic advantage avoiding scars in the visible front part of the abdomen. The laparoscopic as well as robotic-assisted approach before open surgery could be performed safely for the majority of surgical operations nowadays. Laparoscopic and robotic-assisted minimally invasive surgery should be preferred over open surgery due to its advantages [16].

If the robotic approach is not feasible due to any reason, the same technique can also be employed with the laparoscopic approach and the authors executed a similar technique in another cohort of patients using the laparoscopic approach. The major limitations of our study are it being a retrospective analysis and including only a small number of patients.

## Conclusions

Robotic splenectomy using the modified lateral approach can safely be performed by completely avoiding steps of anatomical dissection and restricting the dissection and dissecting close to the spleen which is an advantage in patients with hematological disorders. The operative time, blood loss and overall morbidity of this modified lateral approach without entering the lesser sac are better compared to the published series.
